# Ethyl 6-methyl-8-phenyl-1,2,4-triazolo[1,5-*a*]pyridine-7-carboxyl­ate

**DOI:** 10.1107/S1600536813031152

**Published:** 2013-11-23

**Authors:** Yang Li, Chen Sun, Ran Zhang

**Affiliations:** a School of Chemical Engineering, Taishan Medical University, Taian 271016, People’s Republic of China

## Abstract

In title compound, C_16_H_15_N_3_O_2_, the 1,2,4-triazolo[1,5-*a*]pyridine ring system is almost planar (r.m.s. deviation = 0.0068 Å) and forms a dihedral angle of 61.4 (3)° with the phenyl ring. In the structure, centrosymmetrically related mol­ecules are linked into dimers by pairs of C—H⋯N hydrogen bonds.

## Related literature

For application of [1,2,4]triazolo[1,5-*a*]pyridine derivatives, see: Luo & Hu (2006 ▶); Liu & Hu (2002 ▶). For the synthesis of [1,2,4]triazolo[1,5-*a*]pyridine derivatives, see: Jones & Sliskovic (1983 ▶); Wang *et al.* (2003 ▶); Ge *et al.* (2009 ▶); Jia *et al.* (2010 ▶). For standard bond lengths, see: Allen *et al.* (1987 ▶).
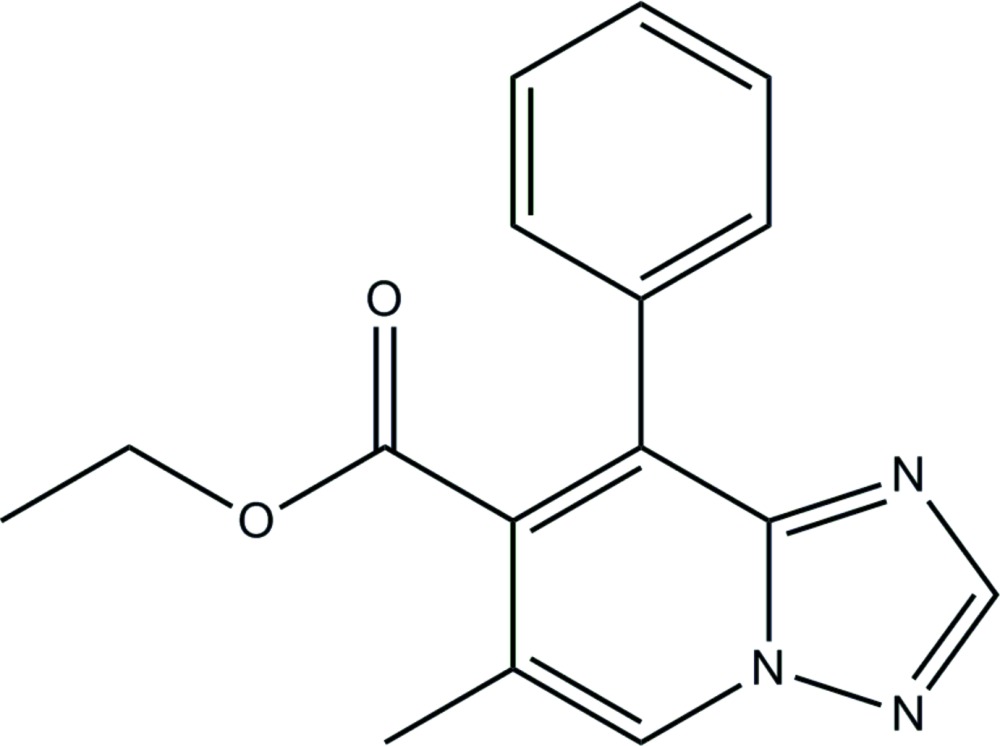


## Experimental

### 

#### Crystal data

C_16_H_15_N_3_O_2_
*M* *_r_* = 281.31
Monoclinic, 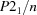

*a* = 13.401 (3) Å
*b* = 7.4825 (19) Å
*c* = 15.068 (4) Å
β = 98.986 (4)°
*V* = 1492.4 (7) Å^3^
*Z* = 4
Mo *K*α radiation
μ = 0.09 mm^−1^
*T* = 298 K
0.23 × 0.19 × 0.15 mm

#### Data collection

Brucker SMART APEXII CCD area-detector diffractometer
Absorption correction: multi-scan (*SADABS*; Bruker, 1999 ▶) *T*_min_ = 0.981, *T*_max_ = 0.987
7312 measured reflections
2611 independent reflections
1920 reflections with *I* > 2σ(*I*)
*R*_int_ = 0.086

#### Refinement

*R*[*F* ^2^ > 2σ(*F* ^2^)] = 0.049
*wR*(*F* ^2^) = 0.152
*S* = 1.05
2611 reflections
192 parameters
H-atom parameters constrained
Δρ_max_ = 0.22 e Å^−3^
Δρ_min_ = −0.23 e Å^−3^

### 

Data collection: *SMART* (Bruker, 1998 ▶); cell refinement: *SAINT* (Bruker, 1999 ▶); data reduction: *SAINT* (Bruker, 1999 ▶); program(s) used to solve structure: *SHELXS97* (Sheldrick, 2008 ▶); program(s) used to refine structure: *SHELXL97* (Sheldrick, 2008 ▶); molecular graphics: *SHELXTL* (Sheldrick, 2008 ▶); software used to prepare material for publication: *SHELXTL* (Sheldrick, 2008 ▶).

## Supplementary Material

Crystal structure: contains datablock(s) 120713d, I. DOI: 10.1107/S1600536813031152/rz5092sup1.cif

Structure factors: contains datablock(s) I. DOI: 10.1107/S1600536813031152/rz5092Isup2.hkl

Supplementary material file. DOI: 10.1107/S1600536813031152/rz5092Isup3.cml

Additional supplementary materials:  crystallographic information; 3D view; checkCIF report

## Figures and Tables

**Table 1:** Hydrogen-bond geometry (Å, °)

*D*—H⋯*A*	*D*—H	H⋯*A*	*D*⋯*A*	*D*—H⋯*A*
C6—H6⋯N2^i^	0.93	2.49	3.332 (3)	151

Symmetry code: (i) 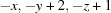.

**Fig. 1.**
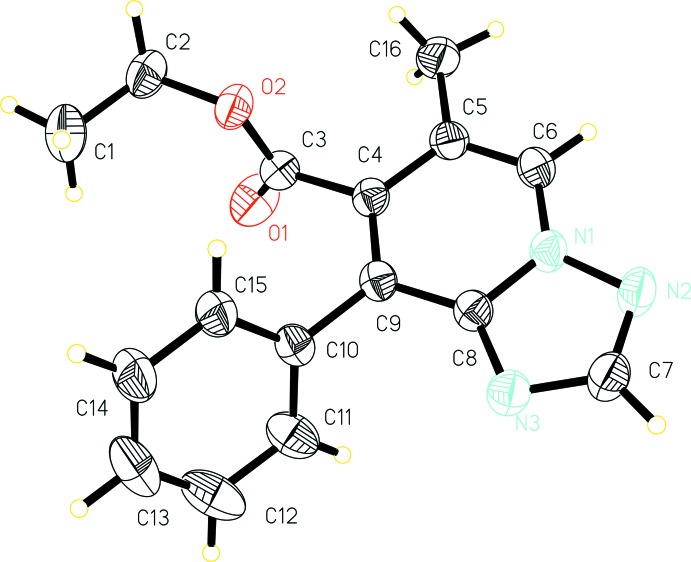
The molecular structure of the title compouns, with displacement ellipsoids drawn at the 30% probability level.

## supplementary crystallographic information

### 1. Comment

[1,2,4]Triazolo[1,5-*a*]pyridine derivatives are important heterocyclic compounds which exhibit antifungal, anticancer and anti-inflammatory activities (Luo & Hu, 2006; Liu & Hu, 2002). Despite possessing outstanding biological activities, only a few [1,2,4]triazolo[1,5-*a*]pyridines are known. Some commonly used synthetic methods are the annulation of the 1,2,4-triazole ring starting with amino substituted pyridines by a multistep procedure (Jones & Sliskovic, 1983). Previously, imidazo[1,5-*a*]pyridines, pyrazolo[1,5-*a*]pyridines, imidazo[1,2-*a*]pyridines and indolizines had been synthesized by a novel tandem reaction in our group (Wang *et al.*, 2003; Ge *et al.*, 2009; Jia *et al.*, 2010). As an extension of this work, the synthesis of [1,2,4]triazolo[1,5-*a*]pyridine heterocycles through this procedure has been undertaken. We present here the crystal structure of one of such compounds, ethyl 8-phenyl-6-methyl-[1,2,4]triazolo[1,5-*a*]pyridine-7-carboxylate.

In title compound (Fig. 1) the the [1,2,4]triazolo[1,5-*a*]pyridine ring system is almost planar, as indicated by its r.m.s. deviation of 0.0068 Å and by the dihedral angle of 0.9 (3)° between the pyridine and triazole rings. The C10–C15 phenyl ring is tilted by 61.4 (3)° with respect to the mean plane through the fused-ring system. All bond lengths (Allen *et al.*, 1987) and angles in the molecule are normal. In the crystal structure, centrosymmetrically related molecules form dimeric units by a pair of C—H···N intermolecular hydrogen bonds (Table 1).

### 2. Experimental

Phenyl(1*H*-1,2,4-triazol-5-yl)methanone (6 mmol), ethyl 4-bromo-3-methylbut-2-enoate (12 mmol), potassium carbonate (1.8 g, 13.2 mmol) and DMF (30 ml) were added to a 100 ml round-bottomed flask and stirred for 8 h. The mixture was then poured into water (200 ml) and extracted with dichloromethane (3 × 50 ml). The organic layers were combined and dried over anhydrous Na_2_SO_4_, then filtered, and the mixture concentrated by rotary evaporation. The crude products were depurated by using column chromatography in 72% isolated yield. Crystals suitable for X-ray diffraction analysis were obtained by slow evaporation of a solution of the title compound in a hexane/ethyl acetate mixture (3:1 *v*/*v*) at room temperature over a period of one week.

### 3. Refinement

All H atoms were found on a difference Fourier map, with C—H = 0.93–0.97 Å and included in the final cycles of refinement using a riding model, with *U*_iso_(H) = 1.2*U*_eq_(C) or 1.5*U*_eq_(C) for methyl H atoms.

### Crystal data

**Table:**

C_16_H_15_N_3_O_2_	*F*(000) = 592
*M**_r_* = 281.31	*D*_x_ = 1.252 Mg m^−^^3^
Monoclinic, *P*2_1_/*n*	Mo *K*α radiation, λ = 0.71073 Å
Hall symbol: -P 2yn	Cell parameters from 3034 reflections
*a* = 13.401 (3) Å	θ = 2.7–25.1°
*b* = 7.4825 (19) Å	µ = 0.09 mm^−^^1^
*c* = 15.068 (4) Å	*T* = 298 K
β = 98.986 (4)°	Block, colourless
*V* = 1492.4 (7) Å^3^	0.23 × 0.19 × 0.15 mm
*Z* = 4	

### Data collection

**Table:**

Brucker SMART APEXII CCD area-detector diffractometer	2611 independent reflections
Radiation source: fine-focus sealed tube	1920 reflections with *I* > 2σ(*I*)
Graphite monochromator	*R*_int_ = 0.086
phi and ω scans	θ_max_ = 25.0°, θ_min_ = 1.9°
Absorption correction: multi-scan (*SADABS*; Bruker, 1999)	*h* = −10→15
*T*_min_ = 0.981, *T*_max_ = 0.987	*k* = −8→7
7312 measured reflections	*l* = −17→17

### Refinement

**Table:**

Refinement on *F*^2^	Primary atom site location: structure-invariant direct methods
Least-squares matrix: full	Secondary atom site location: difference Fourier map
*R*[*F*^2^ > 2σ(*F*^2^)] = 0.049	Hydrogen site location: inferred from neighbouring sites
*wR*(*F*^2^) = 0.152	H-atom parameters constrained
*S* = 1.05	*w* = 1/[σ^2^(*F*_o_^2^) + (0.073*P*)^2^ + 0.2662*P*] where *P* = (*F*_o_^2^ + 2*F*_c_^2^)/3
2611 reflections	(Δ/σ)_max_ < 0.001
192 parameters	Δρ_max_ = 0.22 e Å^−^^3^
0 restraints	Δρ_min_ = −0.23 e Å^−^^3^

### Special details

**Table:**

Geometry. All e.s.d.'s (except the e.s.d. in the dihedral angle between two l.s. planes) are estimated using the full covariance matrix. The cell e.s.d.'s are taken into account individually in the estimation of e.s.d.'s in distances, angles and torsion angles; correlations between e.s.d.'s in cell parameters are only used when they are defined by crystal symmetry. An approximate (isotropic) treatment of cell e.s.d.'s is used for estimating e.s.d.'s involving l.s. planes.
Refinement. Refinement of *F*^2^ against ALL reflections. The weighted *R*-factor *wR* and goodness of fit *S* are based on *F*^2^, conventional *R*-factors *R* are based on *F*, with *F* set to zero for negative *F*^2^. The threshold expression of *F*^2^ > σ(*F*^2^) is used only for calculating *R*-factors(gt) *etc*. and is not relevant to the choice of reflections for refinement. *R*-factors based on *F*^2^ are statistically about twice as large as those based on *F*, and *R*- factors based on ALL data will be even larger.

### Fractional atomic coordinates and isotropic or equivalent isotropic displacement parameters (Å^2^)

**Table:**

	*x*	*y*	*z*	*U*_iso_*/*U*_eq_	
N1	0.10885 (12)	0.7854 (2)	0.55820 (10)	0.0547 (4)	
N2	0.09507 (14)	0.9165 (2)	0.61803 (11)	0.0672 (5)	
N3	0.23653 (13)	0.7566 (2)	0.66890 (10)	0.0628 (5)	
O1	0.25306 (14)	0.4080 (2)	0.34386 (12)	0.0912 (6)	
O2	0.14672 (10)	0.22579 (18)	0.39941 (9)	0.0636 (4)	
C1	0.2563 (2)	−0.0255 (4)	0.3838 (2)	0.0930 (8)	
H1A	0.3166	0.0453	0.3874	0.140*	
H1B	0.2629	−0.1306	0.3486	0.140*	
H1C	0.2463	−0.0598	0.4432	0.140*	
C2	0.16849 (19)	0.0806 (3)	0.34085 (16)	0.0752 (6)	
H2A	0.1827	0.1295	0.2845	0.090*	
H2B	0.1098	0.0035	0.3278	0.090*	
C3	0.19373 (14)	0.3795 (3)	0.39345 (12)	0.0555 (5)	
C4	0.16236 (13)	0.5171 (2)	0.45608 (12)	0.0499 (5)	
C5	0.07336 (14)	0.6198 (3)	0.42544 (13)	0.0556 (5)	
C6	0.04891 (14)	0.7524 (3)	0.47858 (13)	0.0589 (5)	
H6	−0.0087	0.8208	0.4609	0.071*	
C7	0.17325 (18)	0.8910 (3)	0.68132 (14)	0.0687 (6)	
H7	0.1843	0.9626	0.7324	0.082*	
C8	0.19436 (14)	0.6907 (2)	0.58965 (12)	0.0509 (5)	
C9	0.22271 (13)	0.5487 (2)	0.53631 (12)	0.0488 (5)	
C10	0.31516 (14)	0.4442 (3)	0.57064 (12)	0.0541 (5)	
C11	0.40784 (15)	0.5273 (4)	0.58803 (16)	0.0791 (7)	
H11	0.4132	0.6496	0.5789	0.095*	
C14	0.3930 (2)	0.1653 (4)	0.62015 (18)	0.0902 (8)	
H14	0.3876	0.0438	0.6315	0.108*	
C15	0.30836 (17)	0.2630 (3)	0.58754 (14)	0.0698 (6)	
H15	0.2457	0.2068	0.5767	0.084*	
C16	0.01047 (17)	0.5868 (3)	0.33544 (15)	0.0759 (7)	
H16A	−0.0466	0.6659	0.3276	0.114*	
H16B	−0.0127	0.4652	0.3322	0.114*	
H16C	0.0505	0.6081	0.2889	0.114*	
C12	0.49383 (18)	0.4263 (5)	0.61947 (19)	0.1016 (10)	
H12	0.5571	0.4802	0.6294	0.122*	
C13	0.4843 (2)	0.2469 (5)	0.6356 (2)	0.1015 (10)	
H13	0.5414	0.1804	0.6575	0.122*	

### Atomic displacement parameters (Å^2^)

**Table:**

	*U*^11^	*U*^22^	*U*^33^	*U*^12^	*U*^13^	*U*^23^
N1	0.0617 (9)	0.0450 (9)	0.0561 (9)	0.0084 (7)	0.0047 (7)	0.0023 (7)
N2	0.0850 (12)	0.0518 (10)	0.0632 (10)	0.0147 (9)	0.0069 (9)	−0.0068 (8)
N3	0.0732 (11)	0.0605 (10)	0.0531 (9)	0.0066 (9)	0.0045 (8)	−0.0003 (8)
O1	0.1124 (13)	0.0857 (12)	0.0881 (11)	−0.0218 (10)	0.0553 (10)	−0.0121 (9)
O2	0.0725 (9)	0.0494 (8)	0.0730 (9)	−0.0023 (7)	0.0240 (7)	−0.0091 (6)
C1	0.1027 (18)	0.0772 (17)	0.1035 (19)	0.0237 (15)	0.0294 (16)	−0.0102 (15)
C2	0.0908 (16)	0.0612 (13)	0.0759 (14)	0.0012 (12)	0.0202 (12)	−0.0175 (11)
C3	0.0584 (11)	0.0556 (12)	0.0530 (10)	−0.0017 (9)	0.0102 (9)	0.0014 (9)
C4	0.0528 (10)	0.0442 (10)	0.0535 (10)	−0.0008 (8)	0.0106 (8)	0.0044 (8)
C5	0.0560 (11)	0.0505 (11)	0.0581 (11)	0.0009 (9)	0.0023 (9)	0.0046 (9)
C6	0.0580 (11)	0.0506 (11)	0.0649 (12)	0.0104 (9)	−0.0006 (9)	0.0054 (10)
C7	0.0871 (15)	0.0588 (13)	0.0587 (12)	0.0090 (12)	0.0066 (11)	−0.0063 (10)
C8	0.0558 (10)	0.0467 (10)	0.0494 (10)	0.0031 (8)	0.0059 (8)	0.0071 (8)
C9	0.0520 (10)	0.0450 (10)	0.0505 (10)	0.0019 (8)	0.0112 (8)	0.0073 (8)
C10	0.0541 (10)	0.0622 (12)	0.0462 (10)	0.0089 (9)	0.0087 (8)	0.0030 (8)
C11	0.0578 (13)	0.0981 (18)	0.0817 (15)	−0.0013 (12)	0.0118 (11)	0.0211 (14)
C14	0.0914 (19)	0.0786 (17)	0.0925 (17)	0.0314 (15)	−0.0107 (14)	0.0037 (14)
C15	0.0719 (13)	0.0598 (13)	0.0730 (14)	0.0130 (11)	−0.0034 (11)	0.0006 (11)
C16	0.0772 (14)	0.0723 (15)	0.0710 (13)	0.0060 (12)	−0.0106 (11)	−0.0051 (11)
C12	0.0531 (13)	0.161 (3)	0.0913 (18)	0.0025 (17)	0.0116 (12)	0.0282 (19)
C13	0.0799 (19)	0.127 (3)	0.0966 (19)	0.0475 (19)	0.0126 (15)	0.0175 (19)

### Geometric parameters (Å, º)

**Table:**

N1—C6	1.358 (2)	C5—C16	1.501 (3)
N1—N2	1.364 (2)	C6—H6	0.9300
N1—C8	1.368 (2)	C7—H7	0.9300
N2—C7	1.316 (3)	C8—C9	1.420 (3)
N3—C8	1.333 (2)	C9—C10	1.488 (2)
N3—C7	1.347 (3)	C10—C11	1.377 (3)
O1—C3	1.192 (2)	C10—C15	1.385 (3)
O2—C3	1.322 (2)	C11—C12	1.397 (4)
O2—C2	1.458 (2)	C11—H11	0.9300
C1—C2	1.482 (3)	C14—C13	1.355 (4)
C1—H1A	0.9600	C14—C15	1.374 (3)
C1—H1B	0.9600	C14—H14	0.9300
C1—H1C	0.9600	C15—H15	0.9300
C2—H2A	0.9700	C16—H16A	0.9600
C2—H2B	0.9700	C16—H16B	0.9600
C3—C4	1.500 (3)	C16—H16C	0.9600
C4—C9	1.366 (3)	C12—C13	1.374 (5)
C4—C5	1.433 (3)	C12—H12	0.9300
C5—C6	1.347 (3)	C13—H13	0.9300
			
C6—N1—N2	126.33 (15)	N3—C7—H7	121.5
C6—N1—C8	124.03 (16)	N3—C8—N1	109.50 (17)
N2—N1—C8	109.64 (15)	N3—C8—C9	132.10 (17)
C7—N2—N1	101.54 (16)	N1—C8—C9	118.40 (15)
C8—N3—C7	102.27 (16)	C4—C9—C8	117.18 (16)
C3—O2—C2	117.72 (16)	C4—C9—C10	124.22 (16)
C2—C1—H1A	109.5	C8—C9—C10	118.60 (15)
C2—C1—H1B	109.5	C11—C10—C15	119.36 (19)
H1A—C1—H1B	109.5	C11—C10—C9	120.41 (19)
C2—C1—H1C	109.5	C15—C10—C9	120.21 (18)
H1A—C1—H1C	109.5	C10—C11—C12	119.5 (3)
H1B—C1—H1C	109.5	C10—C11—H11	120.3
O2—C2—C1	110.81 (19)	C12—C11—H11	120.3
O2—C2—H2A	109.5	C13—C14—C15	119.6 (3)
C1—C2—H2A	109.5	C13—C14—H14	120.2
O2—C2—H2B	109.5	C15—C14—H14	120.2
C1—C2—H2B	109.5	C14—C15—C10	120.8 (2)
H2A—C2—H2B	108.1	C14—C15—H15	119.6
O1—C3—O2	124.74 (19)	C10—C15—H15	119.6
O1—C3—C4	123.47 (19)	C5—C16—H16A	109.5
O2—C3—C4	111.78 (15)	C5—C16—H16B	109.5
C9—C4—C5	122.70 (17)	H16A—C16—H16B	109.5
C9—C4—C3	119.35 (16)	C5—C16—H16C	109.5
C5—C4—C3	117.77 (16)	H16A—C16—H16C	109.5
C6—C5—C4	118.08 (17)	H16B—C16—H16C	109.5
C6—C5—C16	120.25 (18)	C13—C12—C11	119.6 (3)
C4—C5—C16	121.63 (18)	C13—C12—H12	120.2
C5—C6—N1	119.61 (17)	C11—C12—H12	120.2
C5—C6—H6	120.2	C14—C13—C12	121.1 (2)
N1—C6—H6	120.2	C14—C13—H13	119.4
N2—C7—N3	117.05 (19)	C12—C13—H13	119.4
N2—C7—H7	121.5		

### Hydrogen-bond geometry (Å, º)

**Table:**

*D*—H···*A*	*D*—H	H···*A*	*D*···*A*	*D*—H···*A*
C6—H6···N2^i^	0.93	2.49	3.332 (3)	151

Symmetry code: (i) −*x*, −*y*+2, −*z*+1.
